# Analysis using national databases reveals a positive association between dietary polyunsaturated fatty acids with TV watching and diabetes in European females

**DOI:** 10.1371/journal.pone.0173084

**Published:** 2017-03-29

**Authors:** Jason Pither, Amy Botta, Chittaranjan Maity, Sanjoy Ghosh

**Affiliations:** 1 Department of Biology, IK Barber School of Arts and Sciences, University of British Columbia-Okanagan, Kelowna, Canada; 2 Department of Biochemistry, KPC Medical College, Kolkata, West Bengal, India; Florida International University Herbert Wertheim College of Medicine, UNITED STATES

## Abstract

In recent years, dietary polyunsaturated fatty acids (PUFA) have increased in parallel to sedentary behavior and diabetes across the world. To test any putative association between dietary PUFA and sedentary behavior or diabetes in females, we obtained country-specific, cross-sectional data on sedentary activity and diabetes prevalence from European Cardiovascular Statistics 2012. Age and gender-specific, nutritional data from each country were obtained from nutritional surveys as well. Socioeconomic (GDP), physical environment (urbanization index) and climatic confounders were accounted for each country. Upon analysis, we found a strong, positive association between sedentary lifestyle in 11-yr old girls (> = 2 hours of TV/ weekday) and dietary PUFA across 21 European countries. Further, a weak association of dietary PUFA and a strong relationship of per-capita GDP was established with elevated fasting blood glucose [(> = 7.0 mmol/L; or on medication] among 25+ year old adult females across 23 countries in Europe. In summary, we present novel ecological evidence that dietary PUFA is strongly associated with sedentary behavior among pre-teen girls and weakly associated with diabetes among adult women across Europe. In the latter group, per-capita GDP was a significant predictor for diabetes as well. Therefore, we recommend that prospective randomized controlled trials (RCTs) be implemented to evaluate if ubiquitous presence of dietary PUFA and low socioeconomic status are possible confounders when intervening to treat/prevent sedentary lifestyle or diabetes in female subjects in Western nations.

## Introduction

Observational studies have suggested that an improper diet and sedentary behaviour are independent risk factors for chronic diseases like obesity, diabetes and cardiovascular diseases [1, 2]. However, the inter-relationship between diet and physical activity, if any remains unclear. Traditionally, spontaneous physical activity is recognized to be a function of either total caloric intake or the overall macronutrient composition of the diet [3]. However, whether the chemical type of macronutrients alter physical activity remains unknown.

We reported recently that in Canada, increases in dietary fat between the 1970’s and 2000's are attributable to increased consumption of monounsaturated (MUFA) and polyunsaturated fatty acids (PUFA) but not saturated fatty acids (SFA) [4]. Indeed, to protect against cardiovascular diseases, SFA has been deliberately removed from our food supply in favour of MUFA and PUFA [5–7]. In a recent worldwide survey of dietary fat intakes between 1990 and 2010, both North America and parts of Europe demonstrate a higher intake of PUFAs whereas saturated fat intakes remained similar [7]. In this time frame, USA demonstrated a 318% increase of its diabetic population from 6.5 million to 20.7 million patients [8] and European diabetes rates also increased [9, 10]. The incidence of diabetes in the Western population was indeed paralleled by also a rise in sedentary behavior. North American children and youth spend between 40–60% of their waking hours in sedentary pastimes like TV watching, video games etc. [11, 12]. In a recent European study, a third of the children across eight nations in the IDEFICS (Identification and prevention of dietary- and lifestyle-induced health effects in children and infants) survey, demonstrated increased sedentary behavior which correlated the most with increased TV watching as an indicator [13]. Is it possible that sedentary behaviour is caused by dietary unsaturated fats? Indeed, 4–8 yr old children in USA and Canada increasingly consume a diet rich in unsaturated fats [14, 15]. Most recently, we also showed that even sources of saturated fats like butter which are preferred at a younger age in the Western world, now have increasing n-6 PUFA [16]. Therefore, we hypothesized a possible biological link between unsaturated fat diets and rising sedentary behaviour and diabetes.

With its genetically concordant yet nutritionally diverse population [17], the European region provides a more suitable setting to test for such associations. In Europe, populations demonstrate varied PUFA and MUFA intakes. Mediterranean countries such as Greece, Italy and Spain have higher MUFA intakes through olive oil [18], whereas n-6 PUFA consumption in Central and Eastern European countries are the highest [19]. Nevertheless, even in the European context, the relationship between dietary MUFA, PUFA and metabolic outcomes such as diabetes, remains unclear. For example: (i) in a mixed sex population in England, administration of MUFA or PUFA rich diet for 24 days did not influence insulin sensitivity in type 2 diabetic subjects [20], whereas (ii) a study from Spain involving insulin-resistant females reported the opposite finding [21]. Furthermore, in earlier studies, (iii) high n-6 PUFA was positively associated with insulin resistance in the Israeli population [22], whereas (iv) MUFA was shown to improve insulin sensitivity in Spanish women [23]. In contrast, (v) in a recent study, 6-week MUFA diet in a healthy Portugese population did not demonstrate any changes in biomarkers of diabetes [24].

Controlling for confounders like urbanization, per-capita GDP or climatic variables which alter physical activity levels in humans, we show that sedentary behaviour in pre-teen girls in Europe is significantly associated with PUFA intakes. With respect to adult European women, despite PUFA intakes demonstrating an association, per-capita GDP emerged as the only significant predictor for elevated blood glucose. We suggest that more attention be paid to dietary PUFA and socioeconomic status as potential confounders, when planning lifestyle interventions to treat/correct sedentary behaviour and diabetes, at least in female subjects.

## Methods

### Sedentary behaviour, PUFA intakes and weight gain in European children

While the biological effects of MUFA is gender-neutral, a clear sex difference exists with the metabolic effects of PUFA *in vivo*. PUFA desaturation and bioconversion to carcinogenic eicosanoids as well as its oxidative modification is higher in females [25]. Consistent with this notion, reports have linked countries with high PUFA intakes like Israel and the USA, with a high rate of female cancers [26, 27]. In basic research, we also demonstrated significant loss of spontaneous activity and elevated insulin resistance in mice fed PUFA diets irrespective of either total food intake or body weight [4]. Therefore, we focussed our analysis to the female sex, taking advantage of published data concerning European females from across Europe.

TV watching (screen time) is a prime, universal indicator for sedentary behaviour in pre-teens and adolescents [28, 29], especially in Europe [13]. Among adolescents, sedentary behaviour and screen time increases with age [30]. According to a recent analysis of 161 studies, there was an increase of 30 mins of sedentary behavior with each increasing year among adolescents [31]. Because we wanted to see biological effects of diet and not age, we evaluated TV watching data on the youngest females (i.e. 11 years) reported in the European Cardiovascular Statistics 2012 database (https://www.escardio.org/static_file/Escardio/Press-media/press-releases/2013/EU-cardiovascular-disease-statistics-2012.pdf).

A pre-teen age group has the added advantage of being shielded from a well-known effect of advancing age on loss of physical activity among older adolescents or adults [32]. Moreover as girls also rapidly lower their physical activity levels as they age, biological differences due to dietary variations could be lost in girls in a higher age group, even as early as 13 years [33].

Data about national sedentary behaviour patterns (Table 6.5 in [34]) and prevalence of overweight/obesity (Table 10.3 in [34]) among 11 yr old female children were obtained from the most recent version of European Cardiovascular Statistics 2012 [34]. We used 2009/10 data for TV watching, except for Bulgaria and Italy, for which 2009/10 values were missing, but 2005/06 values were provided. For these two countries, we predicted 2009/10 values based on a highly significant model-II regression relating 2009/10 values to 2005/06 values for the other 19 countries; the regression R-square was 0.80, and had slope and intercept estimates of 0.929 (95% confidence limits: 0.73, 1.17) and 0.325, respectively.

Estimates of sex-specific PUFA and MUFA intakes were obtained from a comprehensive report of nutritional intakes in European children [35]. The report assessed both macro and micronutrient analysis as obtained from both local and national surveys and opinions among experts across Europe. The surveys included in the study had to be a) published, b) based on individual dietary intakes, c) with adequate information available to evaluate its accuracy, d) obtained after 1987, e) with a narrow age window to provide age and sex-specific data, f) of a sample size of at least 25, representative of the national population. MUFA and PUFA intake data in female children was obtained from twenty-one such surveys across various ages across Europe (page S175 and S176 for MUFA and PUFA data respectively in [35]). Sedentary behavior, prevalence of overweight/obesity and nutritional data for female children across Europe are also summarized in **Table 1**.

**Table 1:** Prevalence of sedentary behaviour among 11 yr old girls with mean intakes of MUFA and PUFA in this age group across Europe.

Country	% of female children who watch >2 hrs of TV per day‡	% of 11yr old female children overweight and obese‡	Mean MUFA Intake (% E) †	Mean PUFA Intake (% E) †
Austria	37	16.7	13	5.5
Belgium	47.5*	26.7	14.5	6.7
Bulgaria	75.6**	17.9	10.5	11.6
Czech Republic	56	16.8	-	5.8
Denmark	58	15.3	10.5	4.6
Estonia	68	7	12.5	6.5
Finland	58	13	13.7	6
France	42	14.9	-	4.4
Germany	43	17.7	12.8	6
Greece	64	16	16.9	6.6
Hungary	48	25.9	9.6	2.9
Israel	48	-	5.05	12
Italy	72.8**	35.9	12.2	5.6
Netherlands	42	17.9	12.2	6.4
Norway	64	14.7	10.7	5.6
Poland	42	12.4	13.5	9.5
Slovakia	61	16.2	9.4	7.5
Slovenia	60	24.4	9.6	6.4
Spain	69	22.9	16	4.6
Sweden	66	19.5	11	4.6
United Kingdom	55	26.1	11.5	5.2

‡ Data from Nichols et al; European Cardiovascular Disease Statistics 2012., Table 6.5 and 10.3.

† Data from Lambert et.al.; British Journal of Nutrition (2004), 92, Suppl. 2, S147–S211; Tables in pages S175 and S176. E; energy.

- indicates that no value was available for these parameters in these countries.

* represents average of “Belgium (Flemmish) and Belgum (French)”.

** these values were predicted based on a regression of 2009/10 values on 2005/06 values using the other 19 countries (see methods).

### Diabetes, PUFA intakes and weight gain in European women

Insulin resistance can be undetected for years before it turns into overt diabetes. Thus, to gain insights regarding the incidence of diabetes from epidemiological data, we used data about the prevalence of elevated blood glucose in adult females (25+ yrs old). Similar to pre-teen female data, data about prevalence of elevated glucose across Europe (page 113, Table 11.2 in [34]), and prevalence of female obesity (page 104, Table 10.1 in [34]) were obtained from the European Cardiovascular Statistics 2012. Estimates of sex-specific adult intakes of total fat, MUFA or PUFA [page 232, Table 1 in [36]] in adults were obtained from a recent report providing dietary FA intakes among inviduals above 18 years of age across the world [36]. Sample size of respondents from each country on MUFA or PUFA intakes were also obtained from the same table. Data describing the mean dietary fat composition, incidence of obesity and elevated blood glucose (> = 7.0 mmol/L or on medication) of women from specific European countries are presented in **Table 2**.

**Table 2:** Prevalence of elevated blood glucose among 25+ yr old females across Europe with mean adult intakes of MUFA and PUFA.

Country	% of women with elevated blood glucose*†	% of obese women†	Mean MUFA Intake (% E) ‡	Mean PUFA Intake (% E) ‡	Sample size for nutritional data‡
Austria	4.6	20.8	12.5	8	2123
Belgium	6.4	10.2	13.8	6.8	3245
Bulgaria	8.9	19.2	9.9	11.3	860
Czech Republic	9.1	22.3	13	7	7913
Denmark	5.9	11.8	12	5	3151
Finland	6.3	13.5	12.4	6.2	1594
France	4.3	17.6	11.8	3.9	1089
Germany	6.3	21.1	12.8	6.5	1000
Greece	7.9	25.6	22.3	6.6	20942
Hungary	8.5	18.2	11.3	8.9	3077
Israel	8.7	25.7	11	8	3242
Italy	5.4	9.1	12.8	4.8	1461
Ireland	5.6	21.3	12	7	1097
Netherlands	4.1	10.1	12	6.8	2106
Norway	7.7	21	10.8	5.4	2672
Poland	6.9	23.8	15.4	5.2	2893
Portugal	5.7	13.4	12.4	4.9	489
Russia	10.7	21.6	16	9	9098
Slovakia	9.2	5.9	11.9	8.7	4018
Slovenia	8.8	13.8	13	3.9	2183
Spain	8.8	21.4	15.9	5.6	10208
Sweden	6	11	12.5	4.7	1217
UK	5.7	26	11.7	5.9	434

* Age standardized prevalence estimate of raised fasting blood glucose (≥ 7.0 mmol/L or on medication) (%).

† Data from Nichols et al; European Cardiovascular Disease Statistics 2012., Table 10.1 and 11.2.

‡ Data from Harika et.al.; Ann Nutr Metab 63, 229–238, 2013; Table 1; E; energy.

### Socioeconomic and geographical confounds

The relationship between dietary fats and physical activity in humans is potentially confounded by socioeconomic [37], built environment (such as urbanization [38]) and climatic (e.g. sunlight hours, mean temperature [39]) factors. Moreover, restricting analyses to a single population may encompass little variation, thereby limiting power of such analysis. As an example, in North America, where intake of PUFA is extensive [36], the lack of a control population consuming less PUFA may hide a specific effect of PUFA on physical activity. In contrast, Europe provides a suitable system for conducting analysis of such relationships because: (i) European populations are among the most genetically homogenous in the world [17]; (ii) detailed and extensive data about diet and disease are available for many European countries (see below); (iii) dietary PUFA and MUFA intakes vary considerably among European countries, and (iv) so too do socioeconomic indicators and climate. Level of urbanization and associated physical environments, which can deter or encourage physical activity through access to parks, open spaces and use of non-motorized transport wasincluded as a confounding variable in our analyses. We did so using the urbanization index of the countries, as published by the United Nations World Urbanization Prospectus 2011 Revision, which describes the percentage of the total population living in urban areas. Average physical activity has been associated with the average affluence of a nation [37]. To account for this confounder, per capita GDP data were obtained from the 2011 World Bank data in US dollars.

Physical activity has also been associated with a variety of climate and weather-related variables [40]. Wikipedia, which reports data from country-specific meteorological organizations, was the source for the following climate variables, corresponding to countries’ capital cities: their latitudes, average annual sunshine hours, the average July daily maximum temperatures, and mean annual temperature, which is now depicted in **S1 Fig**. Data for each European country are summarized in **Table 3.**

**Table 3:** Socioeconomic and environmental variables across Europe.

Country	Per-capita GDP (USD)	Urbanization Index	Latitude in °N	Annual sunlight hours	Maximum July temperature (in °C)	Mean annual temperature (in °C)
Austria	51131	67.7	48	1804	25.6	10.2
Belgium	47802	97.5	51	1546	23	10.5
Bulgaria	7589	73.1	42	2300	28	11.4
Czech R.	21656	73.4	50	1668	23.3	7.9
Denmark	61304	86.9	56	1539	20.4	8
Estonia	17177	69.5	59	1738	19	5
Finland	50788	83.7	60	1819	22	5.6
France	43811	85.8	49	1662	25	12.4
Germany	45868	73.9	53	1626	24	9.6
Greece	25962	61.4	38	2848	33.4	18.5
Hungary	13983	69.5	48	1988	26.6	10.5
Israel	51948	91.9	31	3397	29	17.5
Italy	33275	68.4	42	2473	30.3	15.2
Ireland	38365	62.2	53	1453	20.2	11
Netherlands	53537	83.2	52	1662	22	10.2
Norway	100575	79.4	60	1668	21.5	5.7
Poland	13776	60.9	52	1571	23.8	8.2
Portugal	23195	61.1	38	2806	27.9	17.5
Russia	13324	73.8	55	1731	24.3	5.8
Slovakia	18066	54.7	48	2038	27.5	10.5
Slovenia	24965	49.9	46	1798	26.5	10.2
Spain	31973	77.4	40	2769	31.2	14.6
Sweden	59594	85.2	59	1821	21.9	6.6
U.K.*	40975	79.6	52	1480	23.2	11.5

*U.K.; United Kingdom.

GDP: World Bank data in US dollars as of 2011.

Urbanization: United Nations World Urbanization Prospectus 2011 Revision.

### Statistical analyses

Data regarding children and women were analyzed separately. All analyses were conducted using R package [41]. We used a Bonferroni adjustement when conducting multiple tests. Pairwise associations between all variables were visually assessed using scatterplots, and were statistically evaluated using Spearman (rank) correlation. For each response variable of interest, we conducted multiple regressions, each time including per-capita GDP, climate variables, and urbanization index as confounders, and MUFA and PUFA as predictors of interest [42].

With N between 21 and 23 countries (depending on analysis), we (i) used latitude as a single proxy for climate variables, as it was strongly correlated with each of mean annual temperature, maximum July temperature, and annual hours of sunlight (**S1 Fig**), and (ii) excluded MUFA as a predictor variable as initial Spearman's correlation analyses revealed no associations with any of the other variables of interest. This strategy further served to increase sample size and thus power, because some countries were missing MUFA data (**Table 1**).

For the analyses of adult data, the dietary report provides sample sizes associated with estimates of MUFA and PUFA intakes [36]. As these sample sizes varied considerably among countries (from 434 in the UK to 20942 in Greece), we conducted weighted regressions, with log-transformed sample sizes providing the weighting factor. Regression assumptions were assessed via standard residual diagnostics.

## Results

### Sedentary behaviour among European 11-year-old girls is associated with dietary PUFA

**Fig 1:**
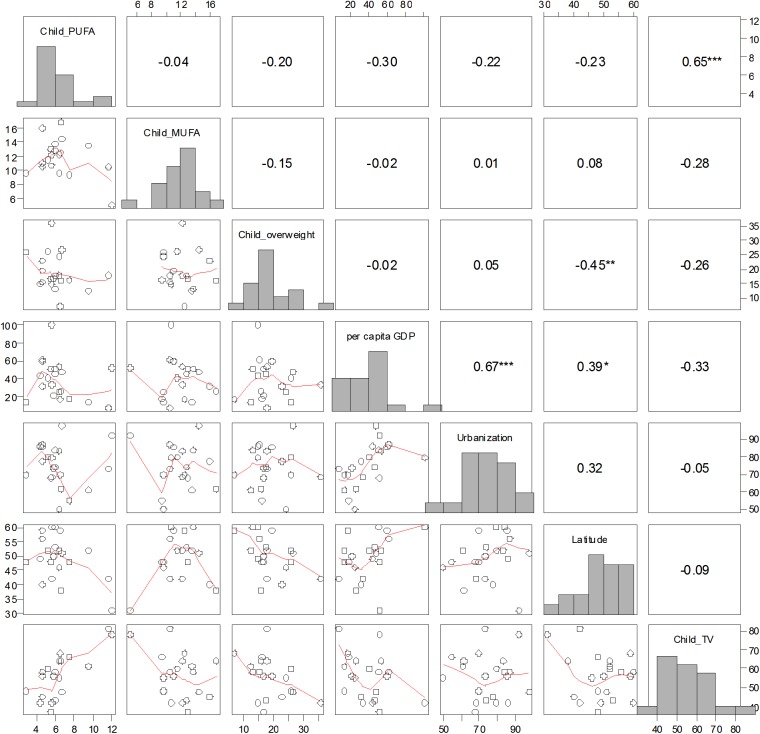
Scatterplot matrix depicting bivariate relationships between all response and independent variables for 11 yr old female children across 21 European countries. For descriptions of variable names and raw data, please see Table 1. Numbers in the upper diagonal represent Spearman rank correlation coefficients (* P ≤ 0.10; ** P ≤ 0.05; *** significant at Bonferroni-adjusted alpha, i.e. P ≤ 0.1 ÷ 21 ≈ 0.005). Lines in lower diagonal panels represent locally weighted smoothers. Histograms of each variable are included in the diagonal.

Sedentary behaviour among European 11-year-old girls was significantly positively associated with reported mean PUFA intake for the specific country (*P*< 0.005; **Fig 1**).Mean MUFA intake was not correlated with any variables. Prevalence of overweight/obesity among European 11-year-old girls was negatively associated with the latitude of the country’s capital city at the unadjusted alpha level (Spearman *rho* = –0.45, N = 20; *P* = 0.045), but not with any other variables (**Fig 1**).

**Fig 2:**
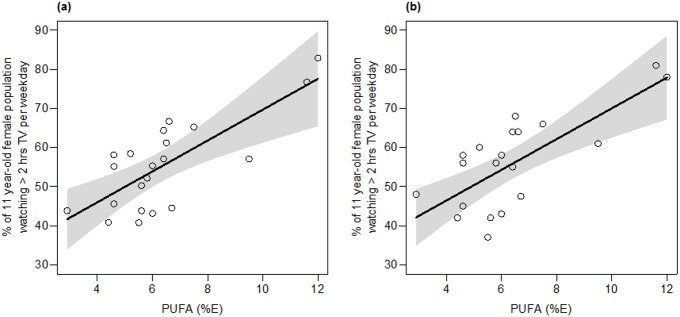
Partial regression plot (a) showing the association between sedentary behaviour of 11 year old girls and mean PUFA, holding all other predictors in the multiple regression constant (N = 21; *P*< 0.001). Grey shading indicates confidence bands around the regression line (see Table 4A for details). Shown in panel (b) is the least-squares regression betweensedentary behaviour of 11 year old girls and mean PUFA, without any other predictors. The regression is highly significant (N = 21; R^2^_adj_ = 0.50; *P* = 0.002).

After accounting for confounders, mean PUFA intake was found to be a highly significant predictor of sedentary behaviour among European 11-year-old girls (**Fig 2A**; **Table 4)**. On average, for every percentage increase in PUFA, and holding all other variables constant, there was a predicted increase of 3.95% in the prevalence of sedentary behaviour (90% confidence limits: 2.25, 5.53). In a least-squares regression that includes only mean PUFA intake as a predictor (thus not accounting for potential confounders), a remarkable 50% of the variation insedentary behaviour is accounted for (**Fig 2B**; regression intercept and slope: 30.71 and 3.93, respectively; F_1,19_ = 21.35, *P* = 0.002).

**Table 4:** Results of multiple least-squares regression for 11-year old European girls. Shown are partial coefficients with 90% confidence intervals (999 permutations). The coefficients can be interpreted as the amount by which the response variable changes when the given predictor (independent) variable increases by one unit, holding all other predictor variables constant. Bold confidence intervals indicate those that exclude zero.

Response variable	Independent variable	Partial coefficient	90% confidence interval
A. % of 11yr old female population that watches 2 hours or more of TV on weekdays	(intercept)	10.73	–26.47, 44.49
	Per-capita GDP (thousands) (US$)	−0.21	−0.33, 0.13
	Urbanization	0.17	−0.09, 0.67
	Latitude of capital city	0.30	−0.31, 0.71
	PUFA (%E)	3.95	**2.25, 5.53**
	Adjusted R-square = 0.52 F_4,16_ = 6.41, *P* = 0.003		
B. % of population as overweight including obesity	(intercept)	48.67	**21.58, 84.73**
	Per-capita GDP (thousands) (US$)	0.02	−0.12, 0.13
	Urbanization	0.10	−0.18, 0.33
	Latitude of capital city	−0.63	**−1.19, −0.21**
	PUFA (%E)	−1.04	−2.25, 0.56
	Adjusted R-square = 0.21 F_4,15_ = 2.26, *P* = 0.111		

In our model of prevalence of overweight among European 11-year-old girls, latitude of the capital city of a country was a significant negative predictor (*P* = 0.02), though the overall multiple regression was not significant (**Table 4**): On average, for every degree increase in latitude of the capital city, and holding all other variables constant, there was a predicted decrease of 0.63% in the prevalence of overweight (90% confidence limits: –1.20, –0.21). Such effects of latitude were predicted earlier on physical activity among school age children and adolescents [40].

### Blood glucose in adult European females and dietary PUFA

**Fig 3:**
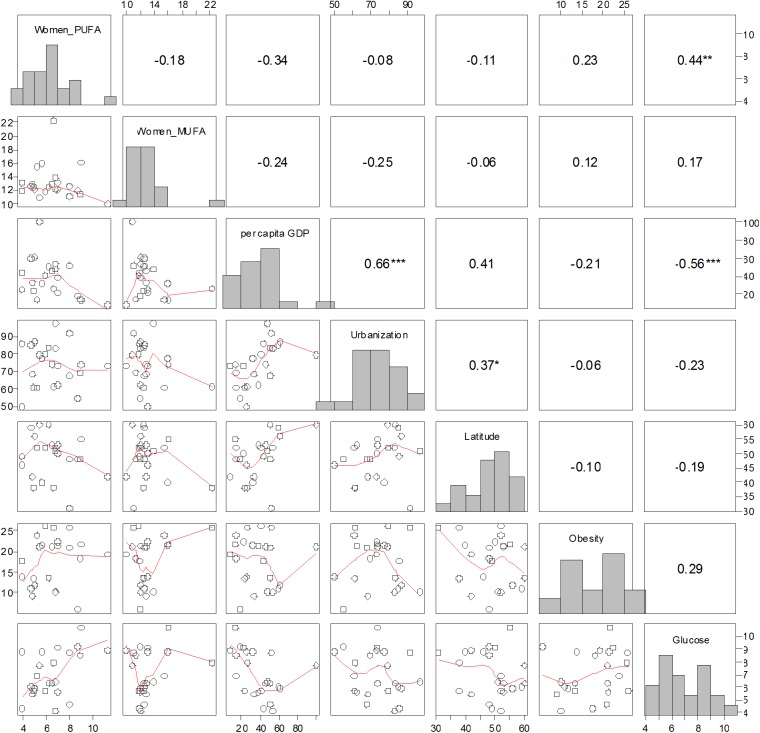
Scatterplot matrix depicting bivariate relationships between all response and independent variables for 25+ years female women across 23 European countries. For descriptions of variable names and raw data, please see Table 2. Numbers in the upper diagonal represent Spearman rank correlation coefficients (* P ≤ 0.10; ** P ≤ 0.05; *** significant at Bonferroni-adjusted alpha, i.e. P ≤ 0.1 ÷21 ≈0.005). Lines in lower diagonal panels represent locally weighted smoothers. Histograms of each variable are included in the diagonal.

Among 25+ year old women, we found that raised fasting blood glucose (> = 7.0 mmol/L) or on medication was positively associated with adult PUFA intakes (**Fig 3**; Spearman *rho* = 0.44; N = 23;*P* = 0.037), but this association was not significant at our Bonferroni-adjusted alpha level of ≈ 0.005. Nevertheless, given our limited sample size (N = 23 countries), we consider this finding noteworthy. Prevalence of obesity among 25+ year old European women was not significantly associated with any of the covariates (**Fig 3**).

In the multiple regression analyses, residual diagnostics revealed Norway to be a strong outlier (due to its extremely high per capita GDP; **Table 3**), so it was excluded from subsequent analyses. Conclusions were qualitatively identical with it included. Per-capita GDP was a significant negative predictor of raised fasting blood glucose (> = 7.0 mmol/L) or on medication in adult women. On average, for every $1000 (USD) increase in per-capita GDP, and holding all other variables constant, there was a predicted decrease of 0.08% in the prevalence of raised glucose (90% confidence limits: –0.120, –0.009) (**Table 5**). Lastly, as observed in our analyses of children data, none of the variables tested were significant predictors of obesity among women (**Table 5**).

**Table 5:** Results of weighted multiple least-squares regression for European women 25 years or older. Shown are partial coefficients with 90% confidence intervals. The coefficients represent the median value of 999 bootstrapped estimates, and can be interpreted as the amount by which the response variable changes when the given predictor (independent) variable increases by one unit, holding all other predictor variables constant. Bold confidence intervals indicate those that exclude zero. The weighting factor was log(sample size) (see Table 2). Norway was removed as an outlier.

Response variable	Independent variable	Partial coefficient	90% confidence interval
A. **% of population with raised glucose levels**	(intercept)	7.29	2.34, 13.64
	Per-capita GDP (thousands) (US$)	−0.08	−0.12, −0.01
	Urbanization	0.03	−0.03, 0.10
	Latitude of capital city	0.02	−0.09, 0.10
	PUFA (%E)	0.20	−0.19, 0.59
	Adjusted R-square = 0.42 F_5,16_ = 4.77, *P* = 0.009		
B. **% of population that is obese**	(intercept)	24.09	2.63, 46.46
	Per-capita GDP (thousands) (US$)	0.02	−0.15, 0.16
	Urbanization	−0.02	−0.26, 0.31
	Latitude of capital city	−0.20	−0.50, 0.24
	PUFA (%E)	0.54	−0.96, 2.13
	Adjusted R-square = 0;F_4,18_ = 0.40, *P* = 0.810		

## Discussion

Over the last 40 years, a synergistic effect of improper diet and lack of physical activity has been blamed for the rapid rise in various chronic diseases like obesity, diabetes and cancer [1]. A high fat diet and sedentary behaviour is considered obesogenic and detrimental. However, the literature remains divided over their inter-relationship. Contrary to common belief, higher caloric intake either does not influence or induces higher spontaneous activity in rodents [43]. In lean humans, excess energy provision is also balanced by increased spontaneous activity and thermogenesis, at least in the short-term [3]. It is increasingly recognized that a low carbohydrate diet (i.e. high in fat) has either no effect on type 2 diabetes in women or is actually beneficial [44–46]. Therefore, rather than total fat, the fat composition of a diet may be vital in influencing sedentary behaviour and diabetes in women.

Besides PUFA and MUFA intakes, in our analyses, we incorporated data from European countries with varied climate (Italy vs. Norway), urbanization (Slovenia vs. England), per-capita GDP (Bulgaria vs.Germany) and geography (Austria vs. Spain). We first demonstrate that sedentary behaviour among 11 year old European female children is significantly positively associated with mean PUFA intakes, after controlling for the significant negative effect of per-capita GDP. Although the underlying biological causes are not clear, there is considerable evidence of clinical depression in women with a high n-6 PUFA diet [47]. Interestingly, depression in teen females is strongly associated with 2 or more hours of TV watching behavior as well [48]. Most recently, we demonstrated that a high n-6 PUFA but not MUFA rich diet, promotes loss of spontaneous activity and insulin resistance in mice [4]. Most interestingly, it has been demonstrated that muscle phospholipid n-6 PUFA content increases with a sedentary profile even in healthy humans [49].

Although sedentary activity data was available, data on diabetes in 11 year was unavailable as insulin or blood glucose values are not usually measured in this age group. As sedentary behavior can be a factor in causing diabetes, we evaluated the incidence of elevated blood glucose in 25+yr old adult females from across Europe. In Spearman’s rank correlation analysis, we found evidence of a positive association between the prevalence of elevated glucose in adult females with reported adult PUFA intakes (**Fig 2**), but given the limited sample size, this association was not significant at our stringent Bonferroni-adjusted alpha level (0.005). Following multiple regression analysis, per-capita GDP emerged as the sole significant predictor of the incidence of elevated glucose in adult females. Socioeconomic status can be a strong predictor of diabetes, and this relationship is stronger for women than in men [50–52]. The probable causes identified involve chronic stress, smoking, poor housing conditions, lack of exercise and education, all of which are related to worsening metabolic profiles. It is also known that low socioeconomic status encourages consumption of cheaper vegetable oils containing high PUFA [53]. In contrast, a direct relationship between breast milk MUFA and higher socioeconomic status is also known [54].

Interestingly, in both young girls and adult women, neither sedentary behaviour nor elevated blood glucose could be associated with the incidence of overweight/ obesity in the population. Despite common perceptions, the relationship between sedentary behavior and obesity is not straight forward in children and adolescents as extensively reviewed earlier [28, 55]. The link between media time and BMI is weak at best in this particular age group. With regards to our analysis, an age of 11 years can also be an insufficient time biologically to accumulate body fat in pre-teen females, even in those with an increased screen time. With respect to adult 25+ yr old females, there is extensive under reporting of obesity in self-report surveys which could have prevented detection of an association [56]. Moreover, with respect to diabetes, it has been known for quite some time that the relationship of adiposity to insulin resistance is confounded in the female sex during reproductive years due to the protective effects of sex hormones [57]. Unfortunately, we feel that the same hormones that prevent against insulin resistance due to general adiposity, often leads to detrimental effects of PUFA specifically by enhancing its bioconversion [25].

PUFA subtypes like n-3 or n-6 PUFAs were not discriminated in our study due to the lack of such differentiations in our databases. At least in the US and most Western nations, both n-3 and n-6 PUFA appear to have increased in recent years with increasing use of vegetable oils like soybean (n-6 and n-3 PUFA at 10 and 50% of total fatty acids) and canola (n-6 and n-3 PUFA at 9 and 21% of total fatty acids) [6, 7]. In a 2015 study, dietary n-3 PUFA was identified as a causative factor for lowering physical activity during class time in iron-deficient South African school children aged 6–11 years, which could not be reversed following iron supplementation [58]. A recent meta-analysis reports that n-3 PUFA supplementation might be related to lowering of aggression in humans which deters spontaneous activity as well [59].

In parallel, dietary n-6 PUFA has been linked to a loss of physical activity in an aged Italian cohort [60]. Regarding diabetes, an example at the population level is illustrated by India. In 2000, there were 31.7 million diabetic cases in India which doubled to more than 62 million individuals within a decade [61]. However, in a national study on the prevalence of diabetes between 1972–1975 involving 35,000 Indians above 14 years of age, diabetes prevalence was only 1.5–2.1% of the population surveyed [62]. Currently, in cities like Kolkata, India, 11.6% of the population suffers from diabetes [61]. It has been speculated for a long time that the rising incidence of diabetes in India is linked to a 'switch' to unsaturated vegetable oils from traditional tropical, saturated oils [63, 64]. In a recent survey involving 27,012 South Indians who traditionally used palm and coconut oils (rich in saturated fats, 9% PUFA) as the chief cooking medium, introduction of sunflower oil (~70% PUFA) was identified as the major cause for developing metabolic syndrome including insulin resistance and hyperlipidemia [65].

### Study limitations

As with any ecological study, associations established using population-level data do not necessarily reflect any causality. In a perfect world, prospective, individual level data from the same year would be obtainable from the entire European cohort in both nutritional intake and sedentary behaviour patterns, which would lead to definitive answers to our question. However, rarely do any one nutritional or behavioral survey cross national boundaries, much less in the same or similar years. The objective of this cross-sectional study was to find associations between PUFA or MUFA intakes and rising sedentary behaviour or diabetes in European populations. To avoid any potential bias on our part, we used a **single** published database of nutrient intake both for adult [36] and children [35]. These are the most up to date, comprehensive nationwide databases available for these age groups available currently. We also used a single database i.e. the European Cardiovascular Statistics database 2012 [22] for sedentary behaviour and blood glucose data.

Overall, this study provides evidence for a positive ecological association between excess dietary PUFA intake across Europe with sedentary behaviour among female children and marginal associations with elevated blood glucose in adult females. A negative association between PUFA intake and per-capita GDP in adult females was also established in this analysis. Considering all confounders, lower per-capita GDP emerged as the strongest predictor for diabetes in adult females. In light of such results, we call for prospective RCTs evaluating a possible link of dietary PUFA and socioeconomic status on inducing sedentary behaviour and insulin resistance in female subjects.

## Supporting information

S1 Fig Climactic relationships across Europe. Scatterplot matrix depicting bivariate relationships between the latitude of the European capital cities, mean annual temperature (MAT), maximum July temperature (July_maxtemp), and total sunlight hours (Sunlight) across European countries. Numbers in the upper diagonal represent Spearman rank correlation coefficients (* P ≤ 0.10; ** P ≤ 0.05; *** significant at Bonferroni-adjusted alpha, i.e.P ≤ 0.1 ÷ 6 ≈ 0.017). Lines in lower diagonal panels represent locally weighted smoothers.Histograms of each variable are included in the diagonal.
(DOCX)
