# Supplementary material for: Analysis using national databases reveals a positive association between dietary polyunsaturated fatty acids with TV watching and diabetes in European females
Source: PLoS One. 2017 Mar 29;12(3):e0173084. doi: 10.1371/journal.pone.0173084 (PMC5371297; doi:10.1371/journal.pone.0173084)
Supplement: S1 Fig — Scatterplot matrix depicting bivariate relationships between the latitude of the European capital cities, mean annual temperature (MAT), maximum July temperature (July_maxtemp), and total sunlight hours (Sunlight) across European countries. Numbers in the upper diagonal represent Spearman rank correlation coefficients (* P ≤ 0.10; ** P ≤ 0.05; *** significant at Bonferroni-adjusted alpha, i.e.P ≤ 0.1 ÷ 6 ≈ 0.017). Lines in lower diagonal panels represent locally weighted smoothers.Histograms of each variable are included in the diagonal. (DOCX) [file pone.0173084.s001.docx]

#### **Supplementary Data**

#### **Analysis using national databases reveals a positive association between dietary polyunsaturated fatty acids with TV watching and diabetes in European females**

Jason Pither, Amy Botta, Sanjoy Ghosh


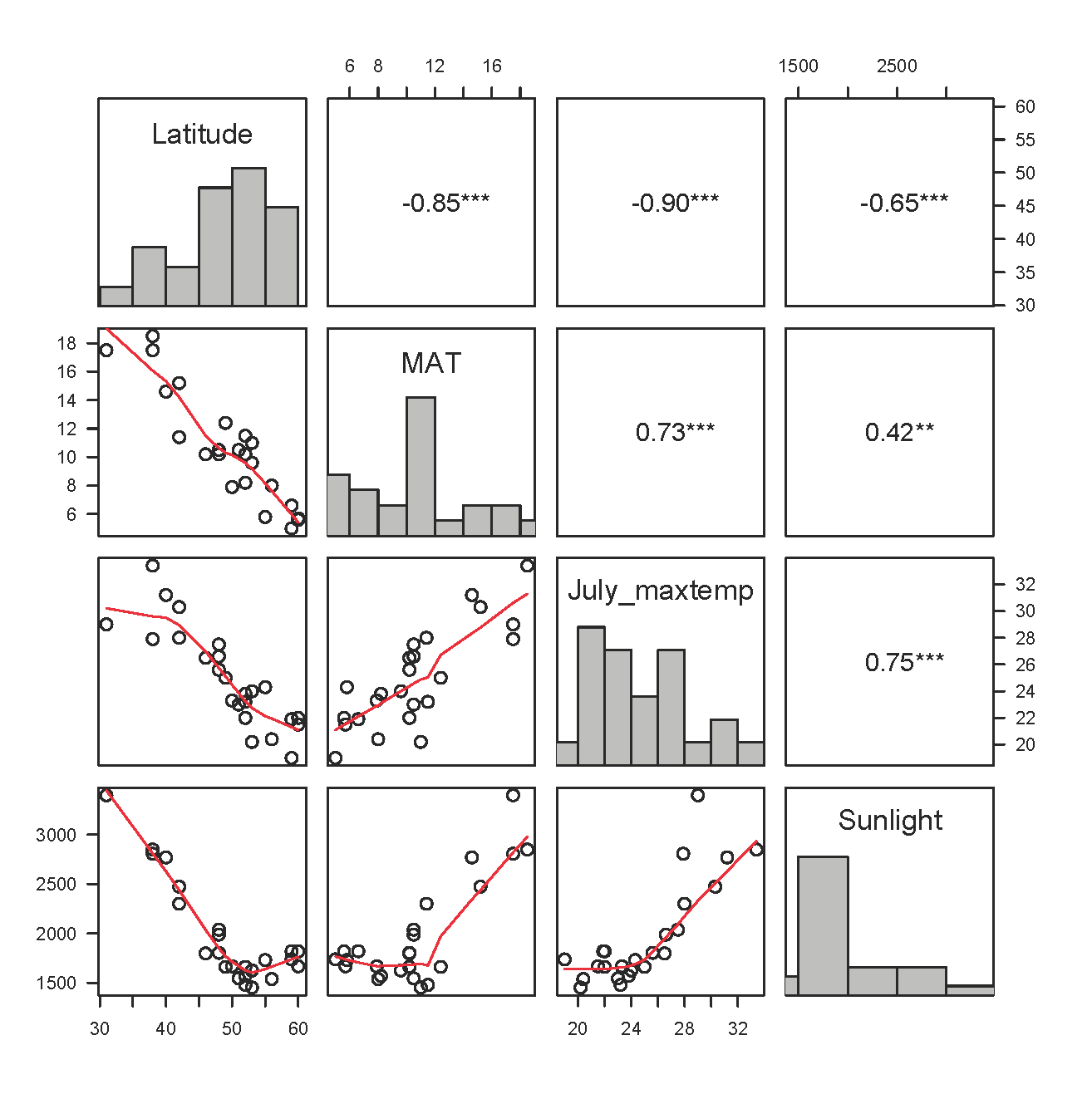


**S1 Figure. Climactic relationships across Europe.** Scatterplot matrix depicting bivariate relationships between the latitude of the European capital cities, mean annual temperature (MAT), maximum July temperature (July_maxtemp), and total sunlight hours (Sunlight) across European countries. Numbers in the upper diagonal represent Spearman rank correlation coefficients (* P ≤ 0.10; ** P ≤ 0.05; *** significant at Bonferroni-adjusted alpha, i.e.P ≤ 0.1 ÷ 6 ≈ 0.017). Lines in lower diagonal panels represent locally weighted smoothers.Histograms of each variable are included in the diagonal.
